# What Can Be Achieved With Motivation-Based Teaching of Medical Students? A Monocentric Retrospective Audit of Retention Among Highly Motivated Graduates Who Underwent the Learning-by-Doing Concept in Anesthesiology and Intensive Care Medicine

**DOI:** 10.2196/10155

**Published:** 2019-04-09

**Authors:** Martina Klincova, Hana Harazim, Daniel Schwarz, Martina Kosinova, Olga Smekalova, Petr Stourac

**Affiliations:** 1 Department of Pediatric Anesthesiology and Intensive Care Medicine University Hospital Brno Faculty of Medicine, Masaryk University Brno Czech Republic; 2 Institute of Biostatistics and Analyses Faculty of Medicine Masaryk University Brno Czech Republic; 3 Department of Anesthesiology and Intensive Care Medicine University Hospital Pilsen Faculty of Medicine in Pilsen, Charles University Pilsen Czech Republic

**Keywords:** problem-based learning, virtual patients, anesthesiology, intensive care, specialization

## Abstract

**Background:**

Medical education, in general, is undergoing a significant shift from traditional methods. It becomes very difficult to discover effective teaching methods within the limited possibilities in patient hands-on education, especially as seen in anesthesiology and intensive care medicine (AIM) teaching. Motivation-based teaching is very popular in all other aspects of education, but it has received scant attention in medical education literature, even though it can make a real difference for both students and physicians.

**Objective:**

The primary aim of this retrospective audit was to find out if proper motivation-based teaching of students via the development of AKUTNE.CZ’s serious games can help retain graduates of the Faculty of Medicine of Masaryk University (FMMU) for the AIM specialty.

**Methods:**

Motivation-based teaching and the learning-by-doing concept were applied to a subject called *Individual Project*. Our topic, *The Development of the Multimedia Educational Portal, AKUTNE.CZ*, has been offered since 2010. The objective has been the development of supportive material in the form of interactive algorithms, serious games, and virtual patients for problem-based learning or team-based learning lectures aimed at acute medicine. We performed a retrospective questionnaire evaluation of all participants from the 2010-2017 period, focusing on their choice of medical specialty in 2017. The data were reported descriptively.

**Results:**

We evaluated 142 students who passed *Individual Project* with topic *The Development of the Multimedia Educational Portal, AKUTNE.CZ* during 2010 to 2017. In this period, they developed up to 77 electronic serious games in the form of interactive multimedia algorithms. Out of 139 students in general medicine, 108 students (77.7%) had already graduated and 37 graduates (34.3%) worked in the AIM specialty. Furthermore, 57 graduates (52.8%) chose the same specialty after graduation, matching the topic of their algorithm, and 37 (65%) of these graduates decided to pursue AIM.

**Conclusions:**

Motivation-based teaching and the concept of learning-by-doing by the algorithm/serious game development led to the significant retention of FMMU graduates in the AIM specialty. This concept could be considered successful, and as the concept itself can also be well integrated into the teaching of other medical specialties, the potential of motivation-based teaching should be used more broadly within medical education.

## Introduction

### Changes in Medical Education

Medical education, in general, is undergoing a significant shift from traditional methods (eg, example, textbooks, lectures, bedside teaching) to a more comprehensive approach using new teaching methods such as problem-based learning (PBL), scenario-based learning (SBL), team-based learning (TBL), and including modern information and communication technology tools such as e-learning, computer simulations, and virtual patients (VPs). The concept of VP itself is quite old but is still existent and initiating discussions about its role in the future of medical education [1-4]. VPs are often used for PBL and SBL concepts, which can have a lot of local particularities depending on their implementation, differing from school to school and variant to variant. Different modifications of these PBL and SBL methods are the current subjects of vast pedagogical researches, including multiple randomized studies [5-13]. A complex Cochrane systematic review on the use of the VP concept in medical education is being prepared. The protocol of the forthcoming review was published recently [14]. The new approach, with more focus on students, especially PBL and SBL using VPs, has been shown to improve the learning skills of medical students and residents over traditional methods [15,16]. Although good evidence in support of a particular education innovation may exist, it is rarely instrumental in decisions to adopt that innovation [17]. Therefore, there is still a wide chasm between student demand for modern and Web-based education and the availability of trained faculty to teach. The design of the learning interface is also important and will significantly affect the learning experience for the student [6,7,13,18]. However, what is often underestimated is the importance of motivation in medical education. We found only 3 relevant studies devoted to this topic [19-21] and it is one of the reasons why we decided to perform this audit.

### Limitations in the Teaching of Anesthesiology and Intensive Care Medicine

Anesthesiology and intensive care medicine (AIM) is a dynamic and time-pressured environment with high demands on firm team communication and leadership, accurate clinical reasoning, and often immediate decision making [22]. Collaborative reasoning occurs in clinical practice but is rarely developed during education [15,23]. The traditional possibilities of practical teaching are mostly very limited because of the inherent nature of the specialty, which requires face-to-face hands-on training [24,25]. Real-time training in critical care units or operating rooms is very problematic for student teaching programs. The lack of time, space, opportunity for learning with mistakes without risk for the patient, and big count of students in one study group make education in this field of medicine trickier. On the contrary, it also gives us a bigger drive for the development of modern, virtual, and educational tools, which together with traditional education and simulation training could be the right concept of education.

### Structure of Anesthesiology and Intensive Care Medicine Teaching at the Faculty of Medicine of Masaryk University

As in most European universities, the Faculty of Medicine of Masaryk University (FMMU) in Brno, Czech Republic, takes only 2 weeks of practice (40 hours) and weekly lectures (half in the fourth and half in the fifth year of study) for AIM teaching in the entire curriculum. For people more interested in this field, there is an option to pass nonmandatory subjects such as anesthesiology, intensive medicine, and pain therapy, each with 1 week of practice (20 hours). In addition, FMMU in cooperation with the educational Web portal, AKUTNE.CZ, offers the unique possibility to focus more on acute medicine within another subject. The teaching subject named *Individual Project* is the obligatory part of the pregradual curriculum of FMMU and is mandatory for registration of final exams. It can be finished at any time between the third and fifth study year. The student has to prove his/her ability to elaborate a student project for the chosen topic under the leadership of the supervisor. *Individual Project* is *terminus technicus*. The student can work on the project alone or in a group of other students but each student is evaluated individually. Since 2010, the topic, *The Development of the Multimedia Educational Portal, AKUTNE.CZ*, has been offered [26]. The objective was to develop supportive material for PBL, SBL, and TBL lectures aimed at acute medicine [27-29]. The supportive material takes an interactive algorithmic form of VP, the development methodology of which is exactly determined (see Study Context in Methods). It aims to develop essential characteristics of any physician dealing with acute patients—algorithmic thinking and correct clinical reasoning. This so-called learning-by-doing concept is motivation-based for both the students and supervisors. It uses the potential of teaching students who wanted to be educated by teachers who want to teach them.

### Highly Motivated Students

We have considered all students who decided to take up our topic in the *Individual Project* subject as highly motivated students. It has been said that it is one of the most challenging topics, especially for its high time requirements. Therefore, only motivated students enroll. This can be an advantage as well as a limitation at the same time (see Discussion). The recruitment of highly motivated students for clinical practice is an important goal for every teacher of pregradual education.

### Objectives

The primary goal was to find out if the proper motivation-based teaching of students can help retain graduates for a certain field of medicine (AIM in our case).

The secondary goal was to find out how big is the concordance between the algorithm topic and postgraduation specialty choice.

The tertiary goal was to find out what percentage of all Czech AIM trainees are graduates who have passed our learning-by-doing concept.

## Methods

### Participants

**Figure 1:**
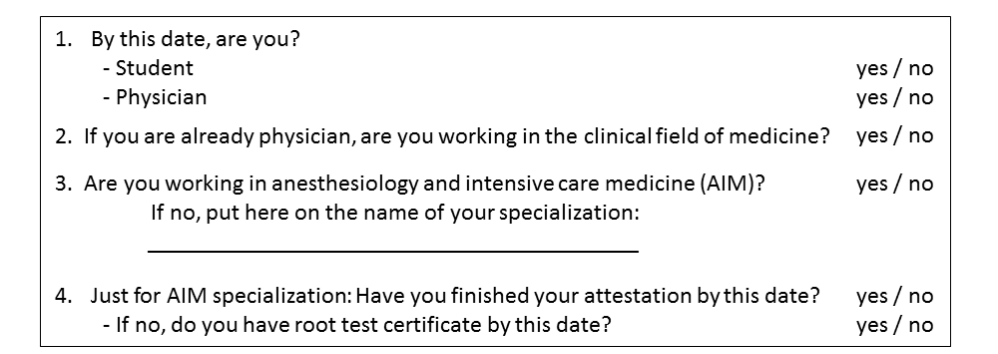
Questionnaire for the students and physicians who passed the topic *The Development of the Multimedia Educational Portal, AKUTNE.CZ* of *Individual Project*, provided by AKUTNE.CZ.

Our study takes the form of a retrospective monocentric audit on the FMMU. For the audit, we invited all the highly motivated students we have worked with to enroll, that is, all students and physicians who have passed our topic in the subject, *Individual Project*, provided by AKUTNE.CZ during the 2010-2017 period. This was the only inclusion criterion for the audit. We contacted all of them via email with a request to answer our questions (Figure 1). All the contacted participants agreed to participate in the audit and data processing. Next, we performed the evaluation of answers focusing on their choice of profession and specialization in medicine in 2017. According to the concept of our study and the subject of evaluation, no ethics committee statement was required after approval by the dean of FMMU.

As a relevant source of medical population statistics data, we used the most recent database available in January 2018, *Czech Republic Healthcare: A Brief Survey of Anesthesiology and Intensive Medicine, 2006-2016,* from the Institute of Health Information and Statistics of the Czech Republic.

The data were reported descriptively using Microsoft Excel 2007 (Microsoft Corporation).

### Study Context

The education portal, AKUTNE.CZ, is an important part of the Medical Faculties Network’s (MEFANET’s) contents [26]. It aims to be a comprehensive source of information and education materials, covering all aspects of acute medicine for undergraduate and postgraduate students of the medical and health professions [29].

The supportive material takes an interactive algorithmic form of the VP (further in the text referred shortly as an algorithm), the development methodology of which is exactly determined [27,28]. Students work in small groups of 2 to 3 members under the supervision of a physician working in the AIM specialization. The estimated time spent on actual work to produce 1 interactive algorithm is approximately between 20 and 100 hours (approximately 2 semesters). The team members devote their time to collaborative work, essential meetings, and self-study. The first draft of an algorithm is in the form of a text document describing the situation at each decision node. The next phase consists of designing both correct and incorrect decision options, inclusive of comments to the correct and incorrect answers. After incorporating the supervisor’s remarks and adding the values of vital signs and physical and laboratory examinations, the whole algorithm is entered node by node into a BackOffice Web application (Institute of Biostatistics and Analyses of FMMU, Czech Republic), together with supplementary multimedia files. Each algorithm must contain 1 picture in each node and at least 1 video, all made by the team members themselves. The resulting algorithm is generated in the form of an HTML5 document, and it is always created in a bilingual version, Czech and English. Before publishing, each algorithm has to undergo an internal review process and, subsequently, it is sent to an external reviewer, experienced clinician, or academic staff member. After incorporating the reviewer’s comments and remarks, the algorithm is supplemented with metadata to be published on the AKUTNE.CZ education portal and indexed by the MEFANET Central Gate [27-29]. All the algorithms are available on the Web and are free of charge for academic use in PBL, SBL, and TBL sessions or e-learning. Each algorithm or even its nodes can be referenced with the use of a URL. For better visualization, see the algorithm workflow schema in Multimedia Appendix 1.

## Results

### Students’ Evaluation

We evaluated all 142 students who passed the *Individual Project* subject with topic *The Development of the Multimedia Educational Portal, AKUTNE.CZ* during the 2010-2017 period. As all 142 students provided us with data, the total number of participants is the same as the number of invited students, that is, 142. In this period, they developed up to 77 electronic VPs in the form of interactive multimedia algorithms. All contacted participants agreed to participate in the audit and data processing.

**Figure 2:**
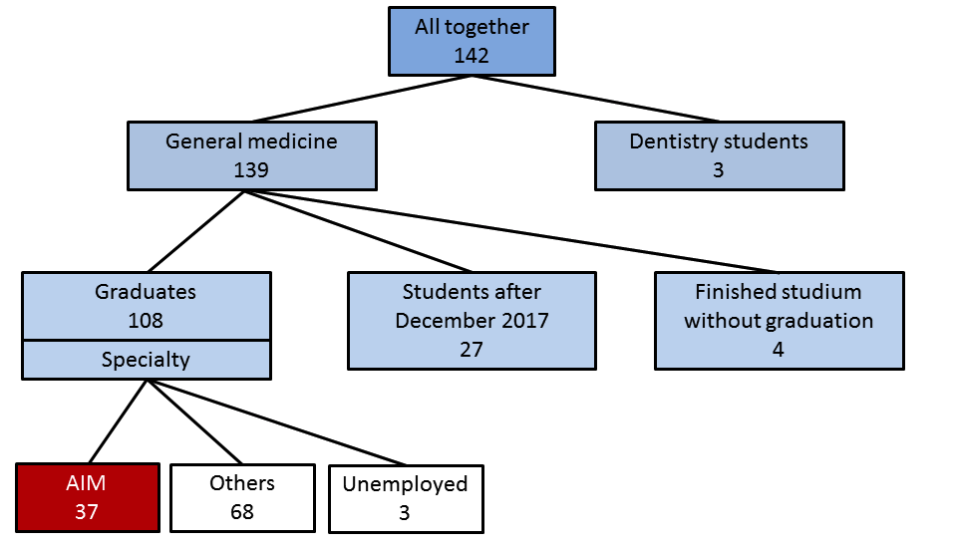
Students who passed *The Development of the Multimedia Educational Portal, AKUTNE.CZ* topic of *Individual Project* during 2010-2017. Numbers are in absolute figures. AIM: anesthesiology and intensive care medicine.

Among the 142 students, 3 students who had studied Dentistry were excluded from further statistics. As depicted in Figure 2, out of 139 students in general medicine, 108 students (77.7%) had already graduated, 27 (19.4%) were still studying (after December 2017), and 4 (2.9%) finished their studies unsuccessfully. In addition, 37 graduates (34.3%) worked in AIM specialization and 68 (63.0%) worked in other clinical fields of medicine. Furthermore, 3 (2.8%) graduates worked and travelled abroad and so were counted as unemployed in medicine.

### Algorithm’s Topics

The topic of each algorithm is dealing with an AIM issue but some of them are multidisciplinary (20 only AIM, 18 internal medicine, 18 surgery and traumatology, 8 gynecology, 6 pediatrics, 3 internal and pediatrics, 1 surgery and gynecology, and 3 dentistry). A total of 57 graduates (52.8%) chose more or less the same specialty after graduation, matching the topic of their algorithm. Furthermore, 37 (65%) of these graduates decided to pursue AIM. We see this finding as very interesting as it can support our hypothesis about the relationship between undergraduate motivation and retention in a specific field of medicine after graduation.

### Anesthesiology and Intensive Care Medicine Specialty in the Czech Republic

In 2016, there were 41,600 physicians in the Czech Republic; AIM employed 2207 (5.30%) of them. A total of 525 (23.79%) were trainees without a finished AIM specialization. Approximately one-third of highly motivated students, after completing their studies, selected AIM as their specialization and helped to expand the number of AIM physicians in the Czech Republic. Since 2010, our graduates accounted for 7.04% of all AIM trainees in the Czech Republic. Presently, 4 of them have already finished their specialty training and 16 have the root test certificate (fulfilling AIM specialty in the Czech Republic takes at least 5 years).

## Discussion

### Principal Findings

To the best of our knowledge, this is the first study focused on the motivation of medical students to be an anesthesiologist using the learning-by-doing concept.

There is no doubt that modern, interactive, multimodal learning and teaching methods are needed in medical education [9,16,17,30,31]. Moreover, motivation-based teaching uses the potential of teaching students who want to be educated by teachers who want to teach them. It is important to evaluate how successful and effective these new methods can be as the importance of motivation is often underestimated [19]. The choice of medical specialty after graduation is certainly multifactorial. However, if we assume that all graduates deal with the same issues (vacancy, salary, and place for living) when looking for a job, then the leading factors for the choice of medical specialty would be enthusiasm and inner feeling of suitability for a certain field of medicine. Enthusiasm is strongly influenced by motivation-based teaching. The learning-by-doing concept provides insight into a certain subject and helps answer the question of personal suitability. Therefore, the retention of highly motivated students in some field of medicine could be an appropriate indicator of the success of the motivation-based teaching concept. The topic, *The Development of the Multimedia Educational Portal, AKUTNE.CZ,* in the *Individual Project* subject is mainly focused on AIM. According to the results, 37 (34%) graduates chose AIM as their further specialization. However, if we take only the group of 57 graduates whose specialty choice is matching the topic of their algorithm, then 37 (65%) of them chose AIM as their specialty after graduation. As there are 43 specialties in the Czech Republic, retention of 65% of graduates for only 1 specialty does not appear as a coincidence. We are aware of the limitations (see Limitations); however, this may indicate that our method of motivation and teaching is effective and successful enough not only to attract motivated students but also to retain them in a certain field of medicine.

Our VPs are content-rich interactive algorithms and their development requires systematic work. The algorithm development itself, as a part of the learning-by-doing process, supports critical thinking and decision making and these are the 2 key skills that are useful, especially for AIM physicians. The creation of such algorithms is extremely demanding, time consuming, and often accompanied by ambiguity and hesitation. The algorithm team meets regularly to check the progress and workflow according to the timetable. A settled schedule motivates students to work systematically and helps them to develop the sense of time management. There are 3 group meetings of the entire AKUTNE.CZ team in a semester to motivate, simulate a creative environment, and provide an open-minded discussion forum. The whole team is built on positive motivation. If students are more interested in AIM, we provide them with the possibility to visit different conferences and other educational events for specialists to gain better insight on our field of medicine. Moreover, AKUTNE.CZ supports a contest, *The Algorithm of the Year,* to reward authors of the 3 best algorithms. Another important aspect is teamwork based on a close collaboration between students and the leader, who is always a doctor working in the AIM specialization. It enriches students’ theoretical knowledge of the practical experience that is provided by the leader. This slightly informal microenvironment also provides students with a great opportunity to meet an AIM professional on a different basis and ask questions about the real nature of the AIM specialty, advantages, and disadvantages, which they would probably never consult with the university authority. We understand that this is very important for decision making and self-evaluation of the inner feeling of suitability for the AIM specialty. Finally, the algorithm is published on the AKUTNE.CZ educational portal website. The fact that students actually produce something *tangible* and even help with the development of multimedia learning material for others can further affect their attitude to academic work for evidence-based medicine.

According to our results, 37 (34%) graduates chose AIM as their further specialization and thus formed 7.1% of all young AIM trainees in the Czech Republic. The lack of physicians and their outflow abroad is a negative phenomenon not only in the Czech Republic but also in other developed countries. Therefore, it is very important to encourage qualified physicians. The Czech Republic, as one of the postcommunist countries, cannot offer salaries as compared with other western European countries. These negative economic aspects may perhaps be somehow alleviated by supporting motivation for needed professions. We believe that motivation-based teaching concepts in medical faculties can make a significant contribution to solving problems with the lack of physicians from the very beginning.

### Limitations

We are aware of the limitations of this study. The audit was just monocentric but it is a new concept of teaching, originally used in FMMU and now spread and used for teaching some other medicine faculties in the Czech Republic. We tried to provide an analysis comparing the number of graduates in total and the number of graduates who decided to pursue AIM in the FMMU before 2010 and after the application of our concept. Unfortunately, the faculty is lacking statistical information about the graduates’ specialization choice. The choice of medical specialty after graduation is certainly multifactorial. Financial factors, free positions, the distance of clinic/hospital from living place of graduates, and others certainly affect decision making of the specialty. In addition, for some graduates, this may become the leading factor. Another limitation can be a passive preselection of highly motivated students, who themselves decided to choose our topic in the subject, *Individual Project*. Therefore, it can be assumed that they were already interested in AIM. However, on the contrary, is it not the right group of students who should be motivated and encouraged to retain in the AIM specialty?

Further investigation is definitely needed. We believe that the positive results of a motivation-based education approach can help motivate others to continue to systematically work with undergraduate students.

### Conclusions

Motivation-based teaching and the concept of learning-by-doing led to significant retention of graduates of FMMU in the AIM specialty (overall 34% and 65% in the group of graduates where specialty choice was matching the algorithm topic). Since 2010, our graduates formed 7.1% of all trainees in the AIM specialty in the Czech Republic. This concept could be considered as successful, and as the concept itself can be certainly well interpolated for the teaching of other medical specialties as well, the potential of motivation-based teaching should be used more broadly within medical education.

## Appendix

Multimedia Appendix 1 Algorithm workflow schema.

## Abbreviations

AIM: anesthesiology and intensive care medicine
MEFANET: Medical Faculties Network
FMMU: Faculty of Medicine of Masaryk University
PBL: problem-based learning
SBL: scenario-based learning
TBL: team-based learning
VP: virtual patient
