# Supplementary figures and images for: What Can Be Achieved With Motivation-Based Teaching of Medical Students? A Monocentric Retrospective Audit of Retention Among Highly Motivated Graduates Who Underwent the Learning-by-Doing Concept in Anesthesiology and Intensive Care Medicine
Source: JMIR Serious Games. 2019 Apr 9;7(2):e10155. doi: 10.2196/10155 (PMC6477577; doi:10.2196/10155)

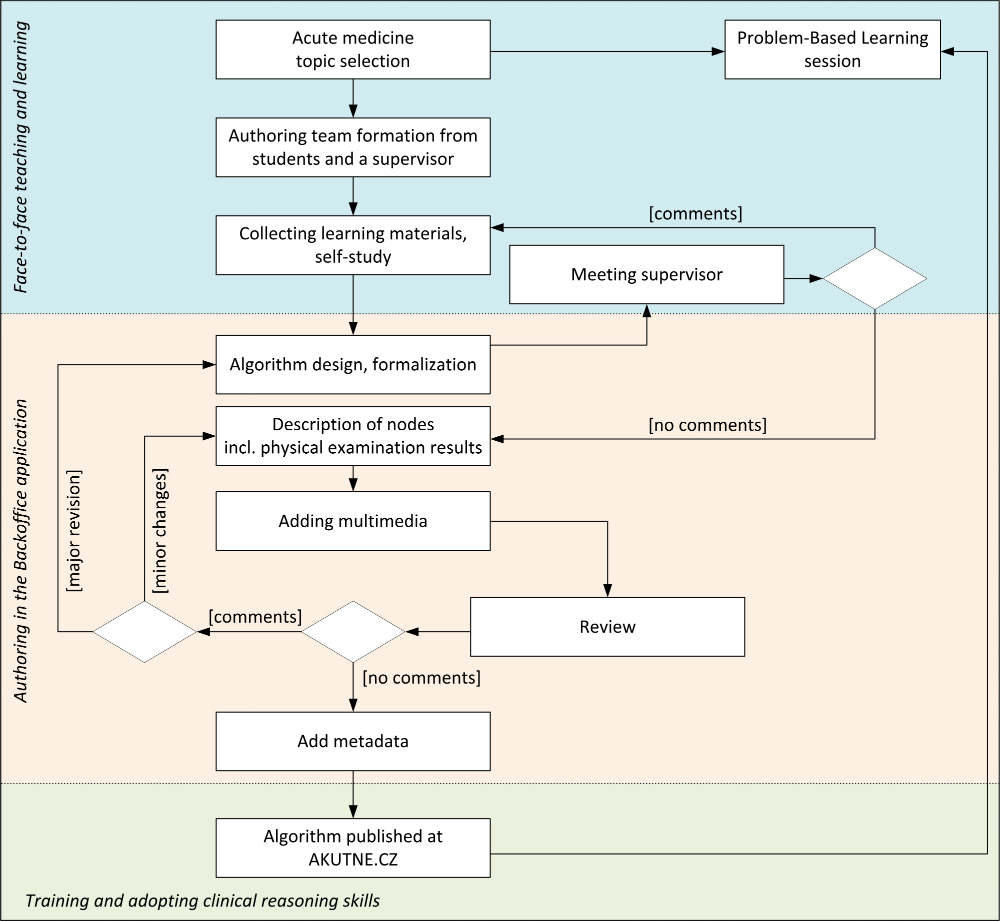

Supplement: Multimedia Appendix 1 [file games_v7i2e10155_app1.jpg]
